# Low‐ to moderate‐level chemical exposures can trigger migraines and are associated with multiple chemical sensitivity

**DOI:** 10.1002/1348-9585.12348

**Published:** 2022-07-20

**Authors:** Luke Curtis

**Affiliations:** ^1^ East Carolina University Brody School of Medicine School of Public Health Hazelwood Missouri USA

To The Editor:

I appreciate the excellent paper on multiple chemical sensitivity (MCS) in migraine patients by Suzuki et al.^1^ This study reported that 19 (20%) of a group of 95 adolescents and adults with chronic migraine met Quick Environmental Exposure Sensitivity Index (QEESI) criteria for MCS.^1^ Among the 19 migraine patients with MCS, the levels of sensory aura, central sensitization, and osmophobia (sensitivity to odors).were significantly higher than for the 76 migraine patients without MCS.^1^

A number of studies have reported that exposure to lower to moderate levels of many chemicals, including exposure to pesticides, organic solvents, and outdoor levels of many pollutants including PM_2.5_, CO, NO_2_, and SO_2_, are associated with increased risk of migraines and other headaches.^2^, ^3^, ^4^

In 1992, Martin and Becker reported on a group of 50 patients who experienced severe headaches from low‐level exposure (43 patients) to high‐level exposure (7 patients).^5^ The most common exposures included solvents in 34 cases, formaldehyde and/or ammonia in 8 cases, and pesticides in 4 cases. The average length of headache following chemical exposures was 4.9 hours. Most of these patients with headache began to experience many repeated headaches and were experiencing severe headaches at much lower levels of chemical exposures than before. Martin and Becker called this syndrome chemical headache exposure syndrome (CHES) and it shares many features with MCS.

Thanks again for the important paper on MCS and migraines. There is a need for much more research on migraines, low‐level chemical exposure, and MCS.

## DISCLOSURE

N/A. This commentary only cited other peer reviewed papers so Ethical Approval, Informed Consent, Registry, and Animal Studies are N/A = Not Applicable.
